# Detection of HLA Antibodies in Organ Transplant Recipients – Triumphs and Challenges of the Solid Phase Bead Assay

**DOI:** 10.3389/fimmu.2016.00570

**Published:** 2016-12-09

**Authors:** Brian D. Tait

**Affiliations:** ^1^Clinical Services and Research, Australian Red Cross Blood Service, West Melbourne, VIC, Australia

**Keywords:** HLA antibody, CDC, ELISA, Luminex, beads, transplantation

## Abstract

This review outlines the development of human leukocyte antigen (HLA) antibody detection assays and their use in organ transplantation in both antibody screening and crossmatching. The development of sensitive solid phase assays such as the enzyme-linked immunosorbent assay technique, and in particular the bead-based technology has revolutionized this field over the last 10–15 years. This revolution however has created a new paradigm in clinical decision making with respect to the detection of low level pretransplant HLA sensitization and its clinical relevance. The relative sensitivities of the assays used are discussed and the relevance of conflicting inter-assay results. Each assay has its advantages and disadvantages and these are discussed. Over the last decade, the bead-based assay utilizing the Luminex^®^ fluorocytometer instrument has become established as the “gold standard” for HLA antibody testing. However, there are still unresolved issues surrounding this technique, such as the presence of denatured HLA molecules on the beads which reveal cryptic epitopes and the issue of appropriate fluorescence cut off values for positivity. The assay has been modified to detect complement binding (CB) in addition to non-complement binding (NCB) HLA antibodies although the clinical relevance of the CB and NCB IgG isotypes is not fully resolved. The increase sensitivity of the Luminex^®^ bead assay over the complement-dependent cytotoxicity crossmatch has permitted the concept of the “virtual crossmatch” whereby the crossmatch is predicted to a high degree of accuracy based on the HLA antibody specificities detected by the solid phase assay. Dialog between clinicians and laboratory staff on an individual patient basis is essential for correct clinical decision making based on HLA antibody results obtained by the various techniques.

## Introduction

Rejection of solid organ allotransplants can be cellular or antibody mediated. In the majority of cases the rejection reaction is directed at human leukocyte antigens (HLAs) expressed on the cells of the transplanted organ. While there is no routine test which can be applied to determine the cellular immune status of potential transplant recipients, the detection of HLA antibodies, particularly those directed at the HLAs of the donor has been at the forefront of donor–recipient histocompatibility testing since transplantation became a clinical reality.

The determination of antibody status is one of the most important investigations that is undertaken in potential organ transplant recipients. While levels of HLA incompatibility can be tolerated due to the quality of immunosuppressive drugs that are now available, the presence of antibodies in the recipient specific for HLA incompatibilities present in the donor can be devastating to the graft.

The first organ transplanted on a routine clinical basis was the kidney and a great deal of lessons we have learned about the impact of HLA antibodies on transplanted organs was learnt during the formative years of clinical renal transplantation.

The pretransplant crossmatch which involves testing the recipient’s serum for cytotoxicity against the donor cells (lymphocytes) was introduced into the testing algorithm in the 1960s in the early days of renal transplantation (1, 2). The test which relies on the detection of complement-dependent cytotoxicity (CDC) is performed in small microtiter trays. The patient’s serum and donor cells are mixed together, rabbit serum as a source of complement is added and lysis due to antibodies in the recipient specific for the donor cells is detected. The crossmatch test is still an essential component of immediate pretransplant testing for all organ transplants and is known as the microlymphocytotoxicity test. A modified form of this test was also used to screen patients’ sera for HLA antibodies and to determine specificity. This method with modifications was the basis of HLA antibody screening for nearly three decades but has been replaced in recent years with more sensitive and reproducible assays of antibody activity. The evolution of HLA antibody testing and the associated laboratory and clinical issues that have arisen with the use of this new technology forms the basis of this review. Although renal transplantation is the basis for many of the lessons we have learned using the new methods of antibody detection, they apply equally to other forms of solid organ transplantation.

## HLA Antibody Detection Assays

### Complement-Dependent Cytotoxicity

The clinical importance of the pretransplant crossmatch and the technology for performing the test was described by Terasaki and colleagues (1, 2) and became known as the microlymphocytotoxicity assay or CDC. Essentially, the test consists of incubating patient serum with potential donor lymphocytes to establish if the recipient has donor-specific HLA antibodies (HLA-DSA). Rabbit serum as a source of complement is added and if HLA-DSA are present lysis of the cells occurs. This lysis can be detected by the original method of dye exclusion or by later developments which included fluorescence. It was quickly appreciated that renal transplant patients with DSA had early hyperacute rejection (3, 4). This test was quickly established as an essential and non-negotiable pretransplant test, a negative result enabling renal transplantation to proceed.

Modifications to the test were made to make the test more sensitive such as prolonged incubation and the use of a second antibody such as an anti-IgG reagent (5) but there remain several technical problems with the test. The assay relies heavily on the viability of the donor cells and in the case of deceased donors optimal viability is not always achievable. In addition to IgG antibodies, the test detects IgM as well as auto antibodies. The latter can be overcome to some extent with the use of 1,4-dithiothreitol (DTT) (6, 7), although this can result in the loss of some IgG antibody (8).

In its original form, the assay was performed using unseparated lymphocytes from peripheral blood, lymph node, or spleen obtained by a gradient separation technique (9). This resulted in the detection of both HLA class I antibodies which react with B and T lymphocytes and also with HLA class II antibodies which react with the class II expressing B cells.

The introduction of cell separation techniques such as ficoll gradient separation (9) with subsequent rosetting T lymphocytes with sheep red blood cells (10) and then later the use of magnetic beads specific for each cell population (11) enabled the distinction to be made between HLA class I and class II antibodies. Other approaches which had varying success were also used.

The main issue with the CDC assay is its sensitivity. The development of more sensitive solid phase assays for antibody detection has basically replaced the CDC approach, but because it is the only functional assay it is still used in many centers as a final test of pretransplant compatibility in the form of the CDC crossmatch. However, even this test is slowly being replaced by the “virtual” crossmatch (see later section).

The CDC assay was modified as an antibody screening technique by using a panel of HLA typed cells and testing each patient’s serum against this panel. The technique is essentially identical to the crossmatch procedure but by using a panel of cells it is possible to determine the HLA specificity of antibodies present. By testing against both T (which express HLA class I) and B lymphocytes (which express both HLA class I and II) it is possible to characterize both class I and class II antibodies when they occur together. An added step of absorbing sera with platelets, which express HLA class I but not class II, prior to testing enables the determination of class II antibody specificity without the added complicating factor of co-occurring class I antibodies (12).

By using a panel of accurate HLA phenotyped cells, it is possible to express the result as a panel reactive antibody (PRA) percentage (i.e., the percentage of cells in the panel giving a positive result) in addition to determining HLA antibody specificities. The PRA is a useful indicator of the probability of a patient giving a negative crossmatch with an unrelated donor.

### Flow Cytometry

The flow cytometry crossmatch (FCXM) was introduced into clinical practise by Garavoy et al. (13). The principle of the test involves incubating donor cells with recipient serum and then adding a fluorescein-labeled second anti-human immunoglobulin antibody that binds to patient antibody bound to the donor cells. The test is read on a flow cytometer, and the degree of positivity is expressed as a channel shift. The main advantage of the FCXM is its sensitivity for antibody detection over the conventional CDC crossmatch (13, 14). In cases where the second antibody is anti-human IgG, it is not possible to discriminate between complement binding (CB) and non-complement binding (NCB) HLA antibodies. However, if that additional information is required, it is possible to use second antibodies to the IgG isotypes and also IgM (15, 16). It is also possible to detect antibodies to both class I and class II HLA antibodies by using markers to differentiate T and B lymphocytes (17, 18).

With the advent of solid phase and in particular bead technology, and the interpretation of weak antibodies detected by those methods the flow crossmatch is used in many centers to assist in clinical decision making. For example, if the flow crossmatch is positive in the case where a weak HLA antibody is detected by the bead assay (19), a decision may be made to invoke a desensitization protocol or to not proceed with the proposed transplant depending on the patient’s transplant and sensitization history. Alternatively, a weak HLA antibody detected by the bead assay in the presence of a negative flow crossmatch may result in the transplant proceeding, but again the patient’s immunological history would be a part of the decision making in such a case.

### Solid Phase Antibody Detection Assays

#### Enzyme-Linked Immunosorbent Assay

The enzyme-linked immunosorbent assay (ELISA) was initially used in the HLA field for detecting levels of HLA both cell bound and free but was adapted for detection of HLA antibodies in serum in 1995 (20).

In the modified assay HLA glycoproteins are immune-precipitated usually from EBV transformed cell lines, and immobilized in the wells of microtiter trays. Sera to be tested are added to the wells and antibodies specific for the HLA molecules bind to the relevant epitope. After washing an anti-human IgG labeled with a reporter molecule such as alkaline phosphatase is then added which binds to the primary anti-HLA antibody molecule. After repeated washing to remove any unbound secondary antibody, a substrate is added which is dephosphorylated by the alkaline phosphatase resulting in a color change.

Two levels of testing are achievable using the ELISA technique. One involves the use of a pool of different class I and class II molecules, which essentially gives a positive or negative result, and the second utilizes HLA molecules derived from single individuals which can be used to determine antibody specificity.

The ELISA technique is more sensitive than CDC in detecting HLA antibodies (21, 22) but has the potential drawback of not distinguishing between complement-fixing and non-complement-fixing antibodies. This assay however has been used as a very effective method for detecting pre- and postsensitization in solid organ transplants (23–25) but has been somewhat superseded by the introduction of fluorescently labeled beads to which HLA molecules have been attached.

#### Luminex^®^ Bead Technology

The introduction of fluorescently labeled beads revolutionized HLA antibody testing during the 1990s. Commercial kits are available (One Lambda, Immucor) which consist of beads impregnated with differing ratios of two fluorochromes resulting in a unique signal for each bead and which have one or several types of HLA molecules attached.

**Figure 1:**
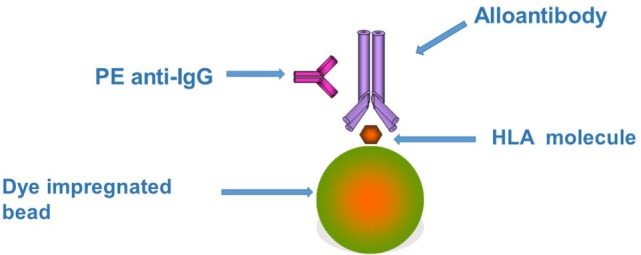
**The figure represents the principles underlying the Luminex bead assay**. Each bead has one or more different types of human leukocyte antigen (HLA) molecules attached depending on the level of testing being performed. If the test serum contains an HLA antibody it will bind to the appropriate HLA molecule. This binding can be detected by the use of a second phycoerythrin (PE)-labeled anti-human IgG. Each bead gives a specific signal when excited by one of the lasers built into the Luminex instrument due to the unique intensity of fluorophore embedded in the bead. A second laser detects the fluorescent excitation produced by the PE on the second antibody. The combination of the two signals indicates first the presence (PE fluorescence) and second the specificity (bead fluorescence) of the HLA antibody in the test serum.

**Figure 2:**
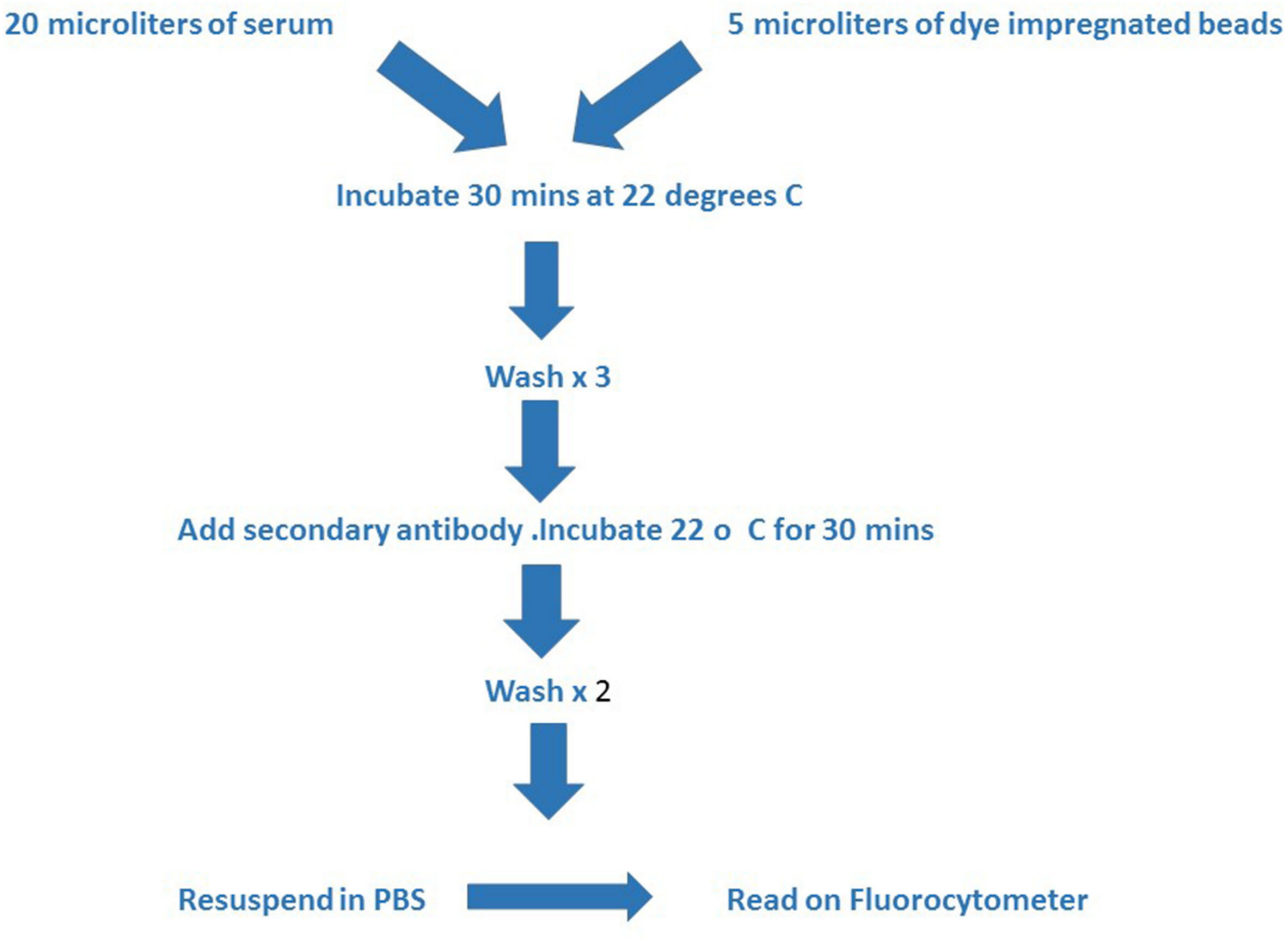
**The figure outlines the technical steps involved in the assay**. The test serum and beads are incubated together at room temperature for 30 min and then washed three times with buffer prior to adding the second antibody. A second incubation period of 30 min at room temperature is followed by two further washes with buffer and then the mixture is resuspended in phosphate buffered saline for reading in the Luminex instrument.

The assay involves first the incubation of a patient’s serum with the beads. If the patient has HLA antibodies the serum will react with the bead expressing the appropriate HLA molecule. After washing, the beads are incubated with a secondary antibody, usually with a phycoerythrin (PE)-labeled anti-human IgG (Figures 1 and 2).

Three levels of testing are possible depending on requirements. The first level provides a positive/negative result with respect to a patient’s antibody status. In this instance, the beads are bound with a large number of class 1 or class 2 molecules derived from lymphoblastoid cell lines. Beads used in second level testing are bound with molecules derived from a single cell line and hence express two HLA molecules for each of the HLA loci (HLA-A, -B, -C for class I and HLA-DR, -DQ and -DP for class II). This testing is essentially analogous to testing with a panel of cells, and therefore, the result can be expressed as a PRA percentage. The third level of testing involves the use of beads bound with single HLA molecules produced by recombinant technology, so called single antigen beads (SAB). These beads provide a real advantage of this technology as complex mixtures of antibodies can be characterized and HLA specificities accurately determined. This technology is now considered essential for the pretransplant testing of sensitized patients.

**Figure 3:**
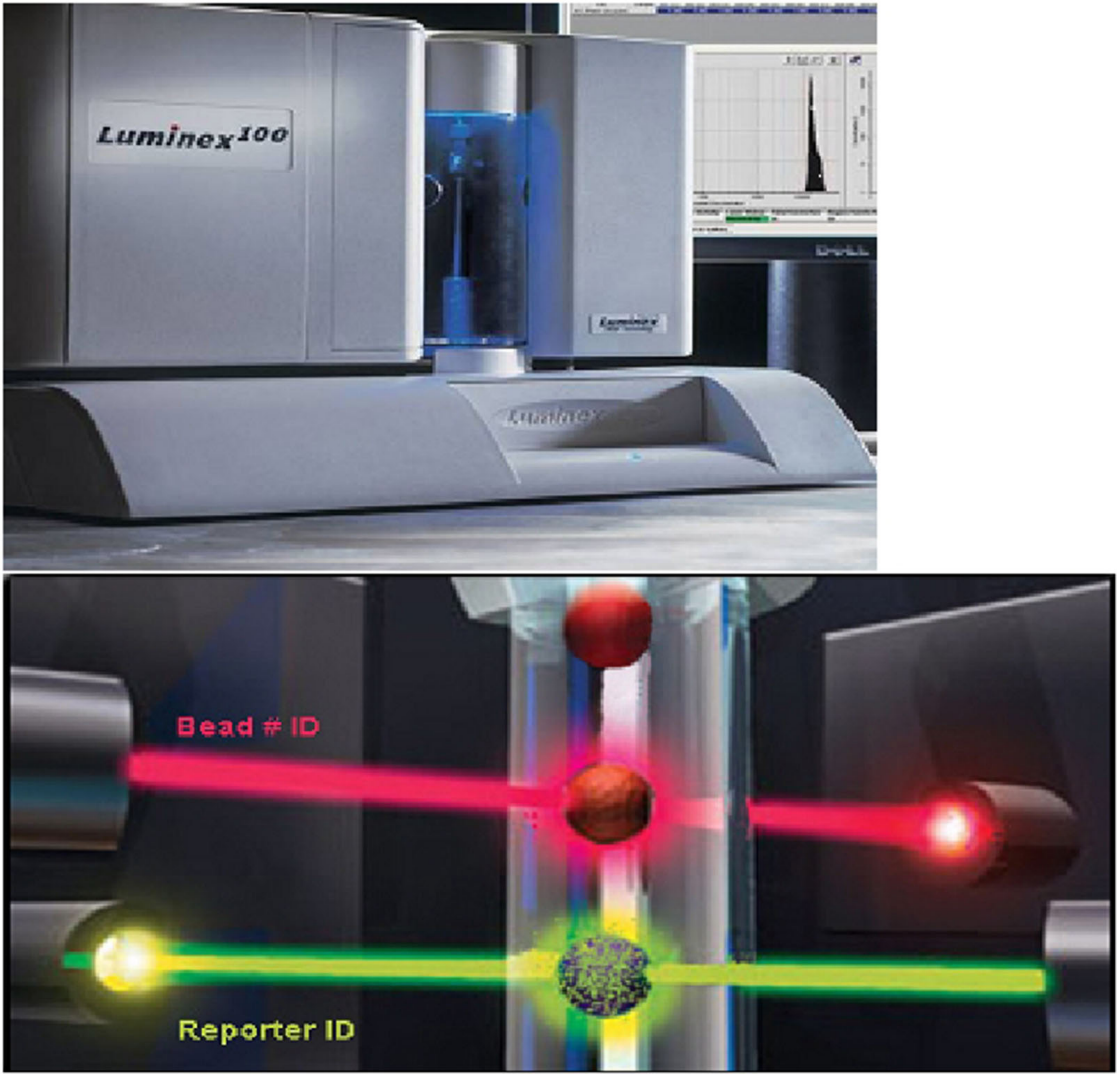
**The top panel shows the Luminex instrument**. There are two lasers in the Luminex instrument (bottom panel). The red laser excites the fluorophore in the bead which provides a unique signal thereby identifying the HLA molecule attached. The green laser excites the phycoerythrin bound to the second anti-human IgG antibody indicating IgG antibody in the test serum has bound to the appropriate HLA molecule attached to the bead. (Modified from a figure provided by Serologicals Corporation.)

There are two common methods for the readout. The first method involves conventional flow cytometry and measuring the channel shift associated with antibody binding. The second which has become the most popular approach is the use of the Luminex^®^ fluorocytometer which utilizes two lasers, one of which excites the fluorochrome in the bead and the other laser excites the PE bound to the detection antibody (Figure 3). The first readout therefore identifies the unique signal of the bead and hence the specificity of the bound HLA molecule, while the second readout indicates whether or not antibody is bound to the specific HLA molecule.

The degree of fluorescence is expressed as a mean fluorescence intensity (MFI), which is normalized by taking into account the degree of fluorescence observed with an antibody negative serum and with beads to which no HLA molecule is attached. A positive control consists of beads bound with PE-labeled human IgG.

## Summary Comparison of Techniques

The advantage of the CDC assay is that it is a functional test involving antibody containing serum and cells. As a crossmatch test it has proved invaluable over the years as a method of avoiding hyperacute rejection due to the presence of HLA-DSA in the recipient (3, 4). As an assay for screening patients for HLA antibodies it has drawbacks. First, it lacks the sensitivity of the other assays described, and second, the assignment of positive and negative reactions can be compromised by viability of the cells used. It also detects both IgG and IgM HLA antibodies in addition to autoantibodies and non-HLA antibodies against other cell surface determinants which have no relevance in organ transplantation.

In the context of organ transplantation, however, it does have the advantage of only detecting CB antibodies. The rationale for replacing this assay with the solid phase assays was driven primarily by the sensitivity issue and the realization that HLA antibodies positive by the solid phase assays but negative by CDC in some cases were clinically relevant (24, 26–29).

Before the introduction of the solid phase assays, the cell-based flow cytometry assay was introduced into clinical practice as a means of providing a more sensitive assay for detection of recipient presensitization to donor-specific HLA. The flow crossmatch however was not amenable to rapid turnaround times and therefore was used primarily in the living related and living unrelated donor situation rather than for cadaveric donors.

The issue of whether the flow assay was as sensitive as the solid phase assays was the subject of debate for some time but the general consensus is that the bead assays are the most sensitive assay for detecting HLA antibodies albeit with their own unique problems (30).

With respect to the two main solid phase assays the fluorescent bead assay has become the gold standard for HLA antibody detection and is now used in most transplant testing laboratories. The remainder of the review will concentrate on the advantages of this technique, and the challenges facing both laboratory workers and transplant clinicians in interpreting the data generated by this assay.

## Advantages of the Luminex^®^ Bead Assay

The Luminex^®^ bead assay is a sensitive method for detection of HLA antibodies and represents the current highpoint in the evolution of HLA antibody detection assays. The additional sensitivity provided by this method has enabled the detection of HLA antibodies in potential transplant patients which are not detectable by other means, particularly CDC (24, 26–29). This increased sensitivity has enabled improvement in the success rate of retransplant patients due to the detection of HLA presensitization as a result of previous grafts and the subsequent avoidance of the relevant HLA specificities on second grafts particularly for DP specificities that are not detected by other methods (31).

The development of SAB has enabled the dissection and specificity determination of complex mixtures of HLA antibodies which is not possible with other techniques. This fine level discrimination coupled with the Matchmaker program (32) has enabled the description of epitope sequences to which HLA antibodies are directed (33–35). Armed with this information consideration of sequences of all alleles regardless of whether or not they are represented on the bead panel allows the identification of all HLA alleles to which a patient is immunized.

Obtaining HLA allele information on potential transplant recipients has permitted the identification of antibodies to alleles within the same antigen group. For example, an A*0301 antibody identified in an A*0302 renal transplant patient (36) and an A*2402 antibody in an A*2403 patient (37). The only coding sequence differences between A*2402 and A*2403 are located at positions 166 and 167 which are the unique substitutions within the epitope recognized by the A*2403 patient. Historically, the presence of A*2403 in a donor would have been considered an antigen match for an A*2402 patient yet it clearly represents a potential immunizing situation.

Identification of antibodies to DQA1, DQB1, DRB3, -4, -5, and DPB1 which is not possible using other antibody screening assays has been enabled by the use of beads containing these molecules. As a result it has become evident that antibodies to DQ and DP (38–42) in addition to DR coexist in organ transplant recipients and have been implicated in negative graft outcomes.

## Interpretive Challenges Associated with the Luminex^®^ Bead Assay

Many of the challenges in interpretation are described in a 2013 report of Consensus Guidelines by an expert Committee under the guidance of The Transplantation Society (43). In addition, reviews have appeared subsequently, which have further contributed to this topic based on more recent data (44, 45). Much of the data had been generated in renal transplantation but the technical issues apply equally to other forms of organ transplantation. The following outlines some of the major issues which require consideration when interpreting bead assay data.

### Sensitivity

Although the bead assay represents the most sensitive method for HLA antibody detection one of the main challenges facing clinicians and laboratory scientists is the interpretation of positive results in the context of a negative CDC crossmatch and/or a negative flow crossmatch, and no indication of presensitization by any other screening technique. The question of the clinical relevance of these HLA antibodies in rejection has been reported in renal, heart, and liver transplantation with mixed results (46–52). Many factors impact on the clinical relevance of these detectable low-level antibodies, one being the MFI cut off for positivity used by the reporting center.

### Mean Fluorescence Intensity

There is no recommended “cut off” value for MFI positivity. Most laboratories set their “cut off” level for positivity based on levels obtained with relevant controls and also on experience gained from clinical results obtained. A useful approach, particularly for multiparous females or previously grafted patients, is to consider each patient on an individual basis. For example, if a previously grafted patient has an MFI level for a particular HLA specificity to which they were exposed on the first graft and the MFI is above the negative values but below the “cut off” level established in the laboratory, this result should be treated with caution. It may indicate a state of presensitization with very low levels of antibody, the production of which can be reactivated on repeat exposure with a second graft bearing that antigen. Such a result may be interpreted differently in a patient with no history of potential HLA preimmunization.

Other factors such as the variable amount of target HLA present on the bead which can be locus and allele specific (53, 54) can result in variation of the MFI obtained. In the absence of an agreed standard for the performance of the Luminex^®^ bead assay, it is incumbent on each testing laboratory to establish their own MFI “cut off” levels in consultation with their clinical colleagues.

### Antibodies to Denatured HLA

The SAB are coated with HLA molecules produced by recombinant technology while the screening beads are coated with HLA molecules immune-precipitated from cell lines. As a result, the SAB express denatured molecules in combination with native molecules. The denatured molecules can express cryptic epitopes not normally accessible by antibody molecules, and it is not possible to distinguish between these two types of antibodies. It would appear intuitive that since the antibody does not have access to the cryptic epitope that these antibodies will not be clinically relevant. However, a need existed for a means of distinguishing between antibodies to denatured and native epitopes. One manufacturer responded by introducing ibeads which are SAB expressing largely native HLA molecules. These beads however were removed from the market in 2014 and the manufacturer recommends as an alternative using an acid wash procedure.

Antibodies to these exposed cryptic epitopes on denatured molecules have been detected in individuals including non-transfused males (55, 56). Studies comparing antibodies to both denatured and native epitopes have demonstrated that the antibodies to denatured epitopes have no clinical impact in renal or heart transplantation (57, 58). Why antibodies to denatured epitopes are found in individuals, particularly non-transfused normal males, is a subject of debate. The concept of cross reactivity with environmental agents such as pathogens or ingested food has been suggested (56).

### Complement-Fixing and Non-Complement-Fixing HLA Antibodies

Unlike the CDC assay which by definition only detects CB HLA antibodies, the bead assay is designed to detect both CB and NCB antibodies. This created debate concerning the NCB antibodies detected by the bead assay, and the concern that many patients were being denied a transplant on the basis of donor-specific NCB antibodies, the clinical significance of which was not established.

Several modifications have been made to the assay to distinguish between CB and NCB HLA antibodies. Using anti-IgG2 and anti-IgG 4 antibodies to detect NCB antibodies Arnold et al. (59) were able to show that up to 40% of re-transplant patients on the waiting list had either HLA class 1 or II NCB antibodies. Wahrmann et al. (60) modified the flow-based bead assay by adding normal serum as a source of complement and anti-C4d as a second antibody and found similar incidence results to Arnold (61).

Modification to the Luminex^®^ method of detection was first described by Chin and colleagues (62). Their approach involved heating the test serum to denature complement and then to add purified human C1q to the serum prior to incubation with the beads. CB antibody binds the C1q and then is detected using a secondary PE-labeled anti-human C1q antibody. This is now the method most commonly used by testing laboratories to distinguish between CB and NCB HLA antibodies. A commercial C1q kit is now available which can detect CB antibodies using either beads in the Luminex system or cells, or for use with cell-based flow cytometry.

The historical association of CDC positive crossmatches with hyperacute or acute rejection led many to believe that CB antibodies detected by the C1q assay would be associated with rejection while NCB antibodies would not. The reality however is that the associations are not so clear cut. Recent studies examining the clinical associations of antibody-mediated rejection with C1q CB and NCB HLA antibodies have yielded some interesting observations.

Calp-Inal et al. (63) showed that the incidence of both acute and chronic rejection was increased in those with CB DSA HLA antibodies pretransplant compared with patients whose DSA were NCB antibodies. Guidicelli et al. (64) demonstrated that while *de novo* CB HLA antibodies were associated with rejection shortly after their appearance NCB antibodies were also associated with rejection in the long term. Piazza et al. (65) also demonstrated a strong association of CB antibodies detected in the C1q assay with inferior graft survival and also demonstrated that the incidence of CB antibodies was increased among those patients with HLA class II antibodies, particularly DQ.

By contrast, Thammanichanond et al. (66) were unable to show a significant effect of CB antibodies with rejection, albeit it in a relatively small cohort of patients. They did however show that the CB antibodies had higher levels of MFI than NCB antibodies.

Likewise, Taylor et al. (67) claim the interpretation of the C1q assay is confounded by the level of antibody, the amount of denatured HLA on the beads and the interference of complement. They further question the justification of its use given the uncertainty in interpretation and the additional cost involved.

There are points to be made with respect to these studies. First, when pretransplant sensitization is involved, in the overwhelming majority of cases the CDC donor crossmatch is negative and therefore lower strength CB antibodies are being selected, which will impact on the overall clinical impact. Second, it is known that CB IgM antibodies which are not detected in the conventional SAB assay can convert to IgG3 CB antibodies posttransplant and are detrimental to the graft (68), which can have a confounding effect on the data analysis when pretransplant antibodies are analyzed. Finally, it appears that NCB antibodies may impact to a degree on graft survival at least in the long term (49, 64).

Recently, a C3d assay has been described (69), similar in principle to the C1q assay, which measures C3d deposition by the addition of human serum to the single bead antigen/antibody complex, followed by the addition of an anti-C3D antibody labeled with PE. Sicard et al. (69) were able to demonstrate in a group of renal transplant patients tested at the time of diagnosis for rejection, patients testing positive for C3d had a higher risk of graft failure. Interestingly, the C1q assay failed to reach statistical significance as a predictor of graft failure.

Comoli et al. (70) in a recent paper presented results of posttransplant testing monitoring for the appearance of *de novo* donor-specific antibodies. Positivity in the C3d assay did not predict graft rejection at the first appearance of *de novo* DSA but at the time of rejection there was a strong correlation. They also demonstrated that conversion within a single antibody from NCB to CB, as demonstrated by C3d positivity, was associated with an increasing MFI. The apparent greater association of rejection with C3d than with C1q may be a reflection of the stage of the complement cascade at which these assays are focused. As indicated by Comoli et al. (70), the presence of C1q does not predict whether the complement cascade will proceed, or just result in C4 deposition on the cell surface. The downstream production of C3d may more accurately predict complete complement activation.

### The Prozone Effect

One of the technical challenges of using SAB for HLA antibody detection is the prozone effect whereby a diluted serum gives a higher MFI than the undiluted serum, suggesting an inhibitory effect which can be abrogated by dilution. One explanation for the inhibitory effect is the presence of IgM antibody of the same HLA specificity blocking the binding of the IgG isotype (71). Since IgM antibodies tend to be a lower titer than IgG the dilution effect was consistent with this interpretation. However, recent research suggests that the effect is due to the inhibitory effect of the C3 component of complement, which is produced as a breakdown product of C1. C3 binds to the beads and inhibits the binding of IgG antibody present in the test serum (72). This problem can be overcome by pre-heating of the test serum to destroy any complement activity or by the addition of ethylenediaminetetraacetic acid (EDTA) to the wash buffer (73) or by the use of dithiothreitol (71) which is also used to eliminate IgM antibodies by disruption of disulfide bonds. The possible confounding effect of prozone should be always considered when interpreting results obtained from using variations of the SAB assay for detection of complement-fixing antibodies.

## The Donor-Specific Luminex Crossmatch

In 2008, Billen et al. (74) reported on the use of commercially available beads for donor-specific crossmatching. The beads are coated with one of two mouse antibodies with specificity for a non-polymorphic sequence on either the class 1 or class 2 molecules. Using lysates of donor cells the beads are able to capture the class 1 and class 2 molecules of the donor which can then be reacted with recipient sera and the bound antibody, if present, labeled with a fluorescently labeled second antibody and read as per the conventional bead assay. Billen et al. compared the results obtained in a group of renal patients with the cell-based FCXM results. They demonstrated a sensitivity using the bead crossmatch of 89% for class 1 and 68% for class 2. Interestingly, they failed to detect antibodies to HLA-DQ and -DP antigens by the bead crossmatch which compromised the value of the LUXM as an alternative B cell crossmatch technique.

Billen et al. (75) further reported on a group of 165 CDC crossmatch negative patients, 32 of whom had bead positive crossmatches. There was no difference in acute rejection free survival when the CDC-bead + crossmatch group were compared with the CDC-bead − crossmatch group. However, the group of patients with bead positive crossmatches due to class 1 antibodies had an inferior long-term 5 years survival (41% compared with 70% for the crossmatch negative group). Positivity for class 2 antibodies had no effect.

Guillaume et al. (76) demonstrated that the LUXM can detect class 1 antibodies with an MFI as low as 2,300 in the SAB technique and 1,300 for class 2. They confirmed the failure to detect HLA-DP antibodies and in addition reported on the failure to detect HLA-C.

Recently, Chaidaroglou et al. (77) reported on a comparison of SAB, FCXM, and LUXM for detection of DSA in a group of heart transplant recipients. They found that there was good agreement between SAB and FCXM but not between LUXM and the other two techniques. They questioned the value of LUXM as a technique for prediction or monitoring.

It would seem based on published data to date that the LUXM cannot be recommended as a stand-alone method for organ allocation.

## The Virtual Crossmatch

The introduction of solid phase assays and the realization that there were a number of patients whose antibodies were detectable by these methods but were negative by the CDC crossmatch cast some doubt on the complete reliance on the CDC crossmatch as a final test of recipient/donor compatibility. Since antibodies with MFI values between 10,000 and 20,000 are required to obtain positive T cell CDC crossmatches in approximately 90% of cases (78), there are clearly some cases where clinically relevant HLA antibodies which are not detected by the conventional crossmatch. The use of specific and sensitive methods for antibody detection and in particular the HLA SAB allowed for the first time a complete picture of the HLA immunization status of individual patients. From this the concept of a “virtual crossmatch” (VXM) was established (79). The VXM takes into account the HLA antibody profile of a patient and predicts which donors will be crossmatch negative. This approach has been used successfully in renal transplantation. Johnson et al. (80) have reported on a large cohort of patients where the final decision to transplant was based on SAB results rather the FCXM result. When analyzed for clinical outcome based on whether the FCXM was positive or negative, despite the fact the FCXM positive group were more “at risk” than the FCXM negative group, the transplant outcomes were comparable, justifying the use of the VXM in preference to the results obtained by FCXM.

Eby et al. (81) have demonstrated the value of the VXM in pancreas transplants as part of the United Network of Organ Sharing in the USA. Pancreata imported from Networks 3 and 4 and transplanted on the basis of VXM had a cold ischemia 5 h shorter than the cases where a FCXM was performed prior to transplant without any compromise in rejection or graft survival incidences.

More centers are expected to rely on the VXM as a prospective guide to the suitability of transplantation as data on the reliability of this procedure is accumulated.

## Use of the Antigen Bead Assay – Lessons Learned

The introduction of solid phase HLA antibody detection methods and in particular the bead assays have revolutionized the clinical management of sensitized patients. However, the introduction of this new technology has posed questions concerning the interpretation of generated data which still require resolution.

The “cut off” MFI values used for assigning positivity vary greatly between laboratories. How much this is due to technical variation and how much is based on correlation with clinical experiences of the local transplant center is difficult to ascertain. Every testing laboratory must determine based on the local performance of the assay and from clinical experience a “cut off” that reflects the level at which antibodies are deemed to be clinically relevant. However in this context, it is imperative to consider the immunological history of the patient. Borderline values or values obtained with antibodies below the “cut off” may be reflective of an increased risk of rejection in patients who have been previously exposed to the particular antigen to which the antibody is directed either by pregnancy or previous grafts. One group of patients of particular interest are those who have undergone desensitization protocols. “Cut off” values therefore should be a guide and not rigidly enforced without careful consideration of the patients’ histories.

The interpretation of HLA antibody results obtained with CB fixing assays requires careful interpretation. The prozone effect needs to be considered in patients who are known to be sensitized but test negative in the CB antibody assays. These patients should in addition be tested at a dilution or treated with EDTA or DTT prior to testing. HLA antibodies which test negative with the CB binding assays should not automatically be dismissed as clinically irrelevant. Although the data suggest in renal transplantation that NCB HLA antibodies are not as damaging to the graft as CB HLA antibodies, and tend to have lower MFI values, there are data which indicate that they do have a lower but nevertheless significant association with rejection. In the absence of convincing clinical data for NCB HLA antibodies in other solid organ transplants, they should also be treated with caution.

The relationship of positive bead assay antibody results with other assays is an important aspect of interpretation. There is universal agreement that an HLA antibody detectable by the bead assay which results in a positive CDC crossmatch is a contraindication to renal transplantation. However, a negative CDC crossmatch in such a situation is less clear cut. The MFI cut off for such a scenario is of the order of 10,000 below which the CDC crossmatch may be negative, but this may vary from laboratory to laboratory based on the sensitivity of the CDC crossmatch and the performance of the SAB under local conditions. Some centers have reported an increased risk of rejection in such patients, others have observed no effect. However, group analyses of such patient groups can mask individual patients whose negative outcome has been influenced by bead + CDC negative antibodies. Some centers recommend the use of a flow crossmatch in such patients. A negative flow and CDC crossmatch may be an indicator that transplantation can proceed. In most cases, this occurs in the presence of a low MFI by the SAB assay. However, the point must again be stressed that the interpretation of multiple assay results must occur in the context of the patient’s history.

In some centers the CDC crossmatch has been replaced with the VXM. The inherent risk in this approach is that some patients will be denied a transplant in what would be a negative CDC situation and with HLA antibodies which may ultimately prove to be not graft damaging. The confounding factors such as prozone and denatured HLA on beads need careful consideration and analysis when relying on virtual crossmatching as the final pretransplant determinant of compatibility.

Published results using the LUXM crossmatch technique to date suggest that there are too many unresolved issues to recommend this technique as a stand-alone method for solid organ transplant allocation.

## Future Directions

The introduction of solid phase assays ushered in a new era in antibody detection both in pre- and posttransplant patients. However, the question of B cell presensitization in patients whose primary antigenic stimulus was years previously and no longer have detectable circulating antibodies still represents a challenge. This problem was referred to briefly in the section on MFI where levels of antibody below the accepted level for positivity in previously grafted patients or multiparous females should be flagged as a potential problem as it may indicate a state of presensitization. Knowing the HLA genotype of previous transplant donors or the biological father of the multiparous patients’ children can be useful in this regard.

Cardiac grafts in multiparous females with a negative CDC crossmatch have a higher incidence of rejection if the donor shares an HLA with the father of the patient’s children (82) even when the primary immunizing pregnancy was up to 30 years previously, demonstrating the long-term effect of memory T and B cells. A comparable study considering HLA epitopes rather than broad antigens has not been reported.

More recently, Mulder et al. (83) used HLA tetramers to study the peptide dependency of HLA antibodies. This technology was utilized by Zachary et al. (84) to investigate the incidence of HLA class I memory B cells in patients with a history of HLA antibodies, but shown to be HLA antibody negative when testing current sera samples. Using tetramers labeled with PE and a labeled CD19 antibody to identify B cells, flow cytometry identified a percentage of cells which bound to the HLA tetramers. The incidence of bound tetramers was significantly greater in previously HLA immunized compared with non-immunized individuals indicating the presence of memory B cells.

A recent report using the ELISPOT assay and HLA class II biotinylated molecules has described the detection of HLA class II-specific memory cells (85) indicating this assay is a useful tool for identifying presensitization in the absence of circulating antibodies (86).

In addition to previously grafted patients and multiparous patients, the use of this technology has potential in monitoring patients who have undergone desensitization protocols prior to transplantation. Depending on the type of desensitization procedure used, it is useful to establish if memory B cells can be detected in patients whose circulating HLA antibody is no longer detected after treatment.

## Conclusion

The introduction of solid phase and in particular bead-based assays, for detection of HLA antibodies has revolutionized clinical management of organ transplant patients. For the first time, laboratory scientists and clinicians are in a position to fully reveal a patients immunological status. This technological breakthrough coupled with HLA sequencing data, which permits the identification of epitopes to which HLA antibodies are directed provides a unique opportunity to maximize the transplant success rate. This new found enthusiasm however is tempered by the fact that there are still areas of both technical and clinical contention which require resolution, such as the role of NCB HLA antibodies and the detection of antibodies to denatured HLA antibodies and their role if any in graft rejection. With this rapid rate of evolution of HLA antibody testing technology, it is imperative that laboratory-based scientists and clinicians communicate on an individual patient basis. Having regard to the immunological history of the patient when interpreting HLA antibody results is critical in maximizing the positive clinical impact of this technology.

## Author Contributions

The sole author was totally responsible for the design and writing of the review.

## Conflict of Interest Statement

The author declares that the research was conducted in the absence of any commercial or financial relationships that could be construed as a potential conflict of interest.
